# Serum Angiopoietin-like Protein 3 Level Is Associated with Peripheral Arterial Stiffness in Patients with Coronary Artery Disease

**DOI:** 10.3390/medicina57101011

**Published:** 2021-09-25

**Authors:** Chien-Hao Hsiao, Yu-Chih Chen, Ji-Hung Wang, Bang-Gee Hsu

**Affiliations:** 1 Department of Internal Medicine, Hualien Tzu Chi Hospital, Buddhist Tzu Chi Medical Foundation, Hualien 97004, Taiwan; allen80413@gmail.com (C.-H.H.); michaelchen@tzuchi.com.tw (Y.-C.C.); 2 Division of Cardiology, Hualien Tzu Chi Hospital, Buddhist Tzu Chi Medical Foundation, Hualien 97004, Taiwan; 3 School of Medicine, Tzu Chi University, Hualien 97004, Taiwan; 4 Division of Nephrology, Hualien Tzu Chi Hospital, Buddhist Tzu Chi Medical Foundation, Hualien 97004, Taiwan

**Keywords:** angiopoietin-like protein 3, peripheral arterial stiffness, brachial-ankle pulse wave velocity, coronary artery disease

## Abstract

*Background and Objectives*: Angiopoietin-like protein 3 (ANGPTL3) is a secretory protein regulating lipid metabolism. This study evaluated the relationship between serum ANGPTL3 level and peripheral arterial stiffness (PAS) in patients with coronary artery disease (CAD). *Materials and Methods*: Fasting blood samples were collected from 95 CAD patients. PAS was defined as left or right brachial-ankle pulse wave velocity (baPWV) > 18.0 m/s by an oscillometric method. Serum ANGPTL3 levels were assessed using a commercial enzyme-linked immunosorbent assay kit. *Results*: Seventeen CAD patients (17.9%) had PAS. Patients with PAS had a significantly higher percentage of diabetes (*p* = 0.002), older age (*p* = 0.030), higher systolic blood pressure (*p* = 0.016), higher fasting glucose (*p* = 0.008), serum C-reactive protein (*p* = 0.002), and ANGPTL3 level (*p* = 0.001) than those without PAS. After multivariable logistic regression analysis, serum ANGPTL3 level (Odds ratio (OR): 1.004, 95% confidence interval (CI): 1.000–1.007, *p* = 0.041) is still independently associated with PAS in CAD patients. The receiver operating characteristic curve for PAS prediction revealed that the area under the curve for ANGPTL3 level was 0.757 (95% CI: 0.645–0.870, *p* < 0.001). *Conclusions*: Serum fasting ANGPTL3 level is positively associated with PAS in CAD patients. Further studies are required for clarification.

## 1. Introduction

Coronary artery disease (CAD) remains a leading cause of mortality and morbidity globally despite improvements in preventive, diagnostic, and therapeutic strategies over several decades [1]. Increased arterial stiffness, characterized by the loss of elastin and increases collagen fibers in arterial walls, is an independent predictor of CAD beyond traditional risk factors such as diabetes, hypertension, dyslipidemia, and smoking [2,3]. It has also showed positive correlation with the severity of CAD extent as well as increased cardiovascular (CV) events and mortality in the CAD population [4,5,6,7]. Brachial-ankle pulse wave velocity (baPWV) is a useful marker for noninvasive determination of peripheral arterial stiffness (PAS) and has also showed good cardiovascular outcome predictions in Asian population [8,9].

Angiopoietin-like protein 3 (ANGPTL3) is a secretory protein mainly produced in the liver and is known to inhibit lipoprotein lipase, an enzyme that degrades circulating triglycerides in the capillaries of muscle and adipose tissue [10]. Individuals carrying ANGPTL3 loss-of-function mutation were reportedly to have low total plasma low-density lipoprotein cholesterol (LDL-C), low plasma high-density lipoprotein cholesterol (HDL-C), and low plasma triglycerides concentrations [11]. Moreover, ANGPLT3 may have a pro-inflammatory and pro-angiogenic effect that implies additional role in the pathogenesis of atherosclerosis [12]. Previously, association between serum angiopoietin-like protein 2 and arterial stiffness was documented in patients with obesity, chronic kidney disease (CKD), receiving maintenance hemodialysis and kidney transplantation [13,14,15,16]. In our previous study, serum ANGPLT3 was positively correlated with the aortic augmentation index in patients with CAD [17]. Because the baPWV is a simple method to assess the arterial stiffness of the medium- to large- sized arteries and the accuracy and reproducibility of its measurement have been confirmed to be acceptable and ANGPLT3 is associated with atherosclerosis. There is scarce evidence about the relationship between ANGPLT3 and PAS measured by baPWV in patients with CAD, we have conducted this cross-sectional study in CAD patients to clarify the association.

## 2. Materials and Methods

### 2.1. Study Design and Participants

Patients with CAD, defined by >50% stenosis in any segment by coronary angiography, was prospectively recruited from August 2020 to December 2020 at a cardiology outpatient department in Hualien, Taiwan. Patients were excluded with an acute infection, acute myocardial infarction, heart failure, aortic aneurysm, dissection, coarctation or malignancy at the time of blood sampling. The medication history in the past two weeks was collected by reviewing medical records. Diabetes mellitus (DM) was defined as two separated blood tests showing either fasting plasma glucose level ≥ 126 mg/dL, or a random glucose level ≥ 200 mg/dL along with classic symptoms of diabetes, or an HbA1c level ≥ 6.5%, or participants using hypoglycemic agents. Hypertension was defined as systolic blood pressure (SBP) ≥ 140 mmHg and/or diastolic blood pressure (DBP) ≥ 90 mmHg or any prescription of antihypertensive drugs in the past two weeks according to the Eighth Joint National Committee (JNC 8) guidelines. The study was approved by the Research Ethics Committee, Hualien Tzu Chi Hospital, Buddhist Tzu Chi Medical Foundation (IRB108-219-A) with informed written consent obtained before study enrollment.

### 2.2. Anthropometric Analysis

All anthropometric measurements were performed by the same operator with the participants in light clothing without shoe. Body weight and height (Tanita WB-380, Tanita Corporation, Tokyo, Japan) were recorded to the nearest 0.5 kg and 0.5 cm, respectively. Body mass index was calculated as the weight in kilograms divided by the height in meters squared [17,18].

### 2.3. Biochemical Investigations

After overnight fasting for 8–12 h, blood samples of approximately 5 mL were obtained from participants and immediately centrifuged at 3000× *g* for 10 min. Serum levels of blood urea nitrogen (BUN), creatinine, triglycerides, total cholesterol, high-density lipoprotein cholesterol (HDL-C), low-density lipoprotein cholesterol (LDL-C), fasting glucose, total calcium, phosphorus and C-reactive protein (CRP) were measured by an autoanalyzer (Siemens Advia 1800; Siemens Healthcare GmbH, Henkestr, Germany) [17,18]. Serum ANGPTL3 (R&D Systems, Inc., Minneapolis, MN, USA) and intact parathyroid hormone (iPTH) levels (Abcam, Cambridge, MA, USA) concentrations were measured using commercial enzyme-linked immunosorbent assay [17,18]. The estimated glomerular filtration rate (eGFR) was estimated from the Chronic Kidney Disease Epidemiology Collaboration (CKD-EPI) equation.

### 2.4. Measurements of Blood Pressure and Brachial-Ankle Pulse Wave Velocity

After blood sampling, the patient was rested in the supine position for ten minutes. The blood pressure was measured for three times by an automatic upper-arm oscillometric device at right brachial artery. The right and left baPWV was assessed by a volume-plethysmographic apparatus (VaSera VS-1000, Fukuda Denshi Co. Ltd., Tokyo, Japan) as previously described [19,20]. The baPWV was calculated by dividing length with pressure wave transit time of an arterial segment between the brachium and ankle. Based on the recursive partitioning analysis in Physiological Diagnosis Criteria for Vascular Failure Committee of Japan, baPWV of 18 m/s was considered to be a statistically adequate cut-off point for individuals with a high risk of cardiovascular disease and subjects with hypertension [21]. So, PAS was defined as left or right baPWV > 18 m/s in this study.

### 2.5. Statistical Analysis

The distribution pattern of the variables was checked using the Kolmogorov-Smirnov test. Continuous variables with normal distribution were expressed as means with standard deviations and tested by the Student’s independent *t*-tests (two-tailed), whereas continuous variables with non-normally distribution were expressed as medians with interquartile ranges and tested by the Mann-Whitney U test. Categorical variables were tested by the chi-squared test. Nonnormally distributed continuous variables were logarithmically transformed when applied to linear regression analysis. Simple linear and multivariate linear regression analyses were used to analyze the relationship between all variables and left baPWV or right baPWV values in patients with CAD. Significant variables correlated with PAS were tested by multivariable logistic regression analysis. A *p* value < 0.05 was defined as statistically significant. All analysis was performed using IBM SPSS Version 19.0 (IBM Corp., Armonk, NY, USA).

## 3. Results

The demographic, clinical, and laboratory characteristics of the patients are shown in Table 1. Of these, 41 (43.2%) had diabetes mellitus (DM), 77 (81.1%) had hypertension, and 17 (17.9%) had PAS. Patients in the PAS group were older (*p* = 0.030), had higher percentage of DM (*p* = 0.002), higher SBP (*p* = 0.016), elevated serum fasting glucose (*p* = 0.008), CRP (*p* = 0.002), and ANGPTL3 (*p* = 0.001) values in comparison to the control group. There were no significant differences between the two groups in terms of sex, hypertension, or anti-hypertension medication used.

**Figure 1:**
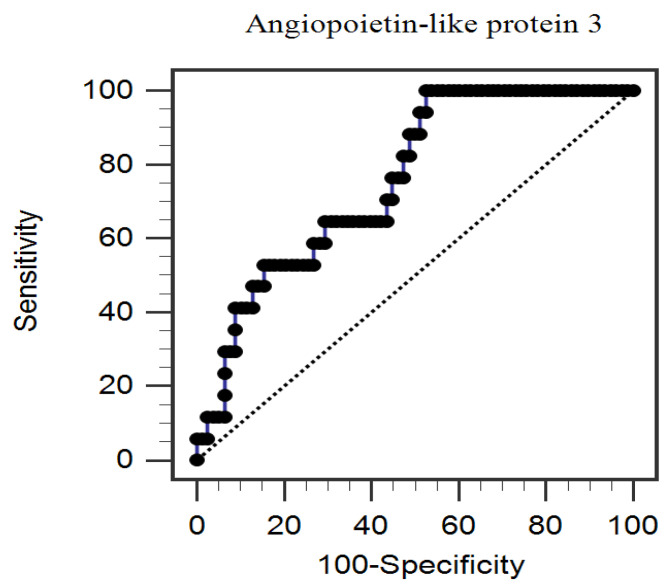
The area under the receiver operating characteristic curve indicates the diagnostic power of serum angiopoietin-like protein 3 levels for predicting peripheral arterial stiffness among 95 coronary artery disease patients.

In the multivariable logistic regression analysis adjusting for factors significantly correlated with PAS, we found that serum ANGPTL3 (odds ratio (OR): 1.004, 95% confidence interval (CI): 1.000–1.007, *p* = 0.041) and age (OR: 1.138, 95% CI: 1.028–1.259, *p* = 0.012) were independently associated with PAS in patients with CAD (Table 2). Furthermore, the ROC curve plotting for PAS prediction revealed that AUC for ANGPTL3 was 0.757 (95% CI, 0.645–0.870; *p* < 0.001) (Figure 1).

**Figure 2:**
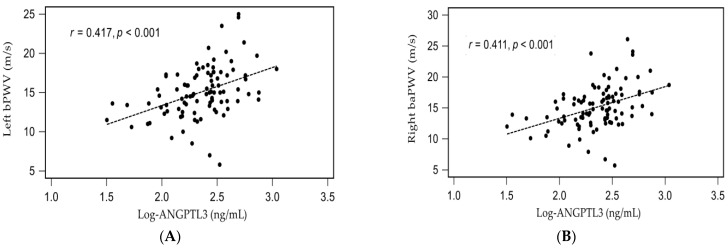
Relationships between logarithmically transformed angiopoietin-like protein 3 (log- ANGPTL3) and (**A**) left brachial-ankle pulse wave velocity (left baPWV), or (**B**) right brachial-ankle pulse wave velocity (right baPWV) among 95 coronary artery disease patients.

The simple linear regression analysis showed that left baPWV value or right baPWV value was significantly positively correlated with age, DM, SBP, as well as logarithmically transformed CRP (log-CRP) and log-ANGPTL3, while negatively correlated with eGFR (Supplementary Tables S1 and S2). Moreover, log-glucose was significantly positively correlated with right baPWV values (*p* = 0.047) by simple linear regression analysis (Supplementary Table S2). After being analyzed by multivariate stepwise linear regression analysis, DM (*β* = 0.236, adjusted R^2^ change = 0.040, *p* = 0.006), age (*β* = 0.190, adjusted R^2^ change = 0.025, *p* = 0.036), log-CRP (*β* = 0.265, adjusted R^2^ change = 0.067, *p* = 0.003), and higher log-ANGPTL3 levels (*β* = 0.286, adjusted R^2^ change = 0.165, *p* = 0.002) were significantly correlated with left baPWV values, while DM (*β* = 0.229, adjusted R^2^ change = 0.046, *p* = 0.008), age (*β* = 0.254, adjusted R^2^ change = 0.048, *p* = 0.004), log-CRP (*β* = 0.270, adjusted R^2^ change = 0.083, *p* = 0.003), and higher log-ANGPTL3 levels (*β* = 0.299, adjusted R^2^ change = 0.160, *p* = 0.001) were significantly correlated with right baPWV values, respectively (Supplementary Tables S1 and S2). To better visualize the results, two-dimensional scattered plots of log-ANGPTL3 level with left baPWV or right baPWV among these CAD patients were drawn, which are presented as Figure 2A,B, respectively.

## 4. Discussion

This study found that CAD patients with increased PAS measured by baPWV had concomitant older age, higher prevalence of DM, higher DBP, higher serum fating glucose, CRP and ANGPTL3 levels. Serum ANGPTL3 level and older age were independently associated with PAS in patients with CAD after multivariable adjustment. After adjustment for confounders, the left or right baPWV values were independently positively associated with DM, age, log-CRP and log-ANGPTL3 levels in patients with CAD.

Aging is a major determinant of arterial stiffness by reducing the elastin and increasing collagen component as a consequence of repetitive stretched and recoil with each heartbeat [22,23]. baPWV value predicts the risk of all-cause mortality in type 2 DM, peritoneal dialysis, and community-dwelling older Japanese [24,25,26] and also is an independent prognostic factor for the cardiovascular death, nonfatal myocardial infarction, coronary revascularization, nonfatal stroke, and hospitalization for cardiovascular causes in patients with CAD [27]. baPWV value has been shown to increase with age and is also a vascular aging marker [22,23]. Consistent with these reports, our results also showed a positive correlation between age and baPWV values in patients with CAD. Furthermore, we similarly found that older age is independently significant predictors for the development of PAS, after adjusting the confounders of CAD patients.

Beyond aging, DM also plays an important role in the pathogenesis of arterial stiffness. First, advanced glycation end products (AGEs), formed between interaction with multiple sugar moieties and the free amino acid residues of proteins, may cause increased collagen component in arterial wall due to pathologic cross-linking with collagen that is resistant to proteolysis [28]. Moreover, insulin resistance contributing to endothelial dysfunction and subsequent dysregulation of nitric oxide and endothelin is associated with higher smooth muscle resting tone and increased arterial stiffness [29]. Blood pressure and chronic kidney disease also are the risk factors that increase baPWV value [21,22]. Chen et al. noted baPWV values are positively correlated with SBP in a 45,092 participants study [30]. Previous reports have shown baPWV values are positively correlated with high-sensitivity CRP in the 45–54 years group and in a male Japanese population [31,32]. In our previous reports, PAS was associated with higher SBP, higher percentage of DM, serum insulin levels, and homeostasis model assessment of insulin resistance in patients who underwent kidney transplantation [20,33]. The positive correlation between SBP, DMlog-CRP, and baPWV values, while negative correlation between eGFR and baPWV values was also showed in our patients with CAD.

ANGPTL3 is a 460-amino acid liver-derived secretary protein, which is composed of an N-terminal coiled-coil domain involved in inhibition of lipoprotein lipase as well as a C-terminal fibrinogen-like domain affecting angiogenesis [10,34]. Lipoprotein lipase releases free fatty acids carried in circulating chylomicrons and VLDLs to the muscle cells and adipose tissue, and its inhibition leads to hypertriglyceridemia that provokes atherosclerotic plaque formation [10,11,34]. Moreover, the activation of integrin α_V_β_3_ by the C-domain of ANGPLT 3 promotes vascular smooth muscle cell migration to the intima, foam cell formation, and inflammatory response, which leads to plaque neovascularization [12]. A meta-analysis of 19 studies involving up to 180,180 individuals showed a 34% reduction of CAD odds ratio among carriers of an ANGPTL3 loss-of function mutation compared to control participants [35]. Similarly, in the DiscovEHR study involving 13,102 patients with CAD and 40,430 control participants, the author reported a 39% lower odds ratio of CAD in ANGPTL3 loss-of-function carriers than non-carriers [36]. The positive correlation between serum ANGPTL3 with PAS was showed in our patients with CAD. Interestingly, CRP was also a positive correlation between PAS in our study which may reinforce the well-known effect of inflammation in the formation of atherosclerosis [37], but the causal relationship between ANGLPT3, inflammation, and atherosclerosis needs further clinical studies for clarification.

There are some limitations in the present study. First, the cross-sectional design with a limited number of CAD patients precludes the confirmation of causality between ANGLPT3 and PAS in patients with CAD. Second, several medications for hypertension and dyslipidemia may affect arterial stiffness through arterial structural and functional alteration and become potential confounders between the association between ANGLPT3 and arterial stiffness [38]. Our results showed that the use of angiotensin-converting enzyme inhibitors, angiotensin-receptor blocker, β-blocker, calcium-channel blocker, fibrate did not significantly differ between control and PAS group. Additionally, we did not evaluate other non-pharmacologic factors that may affect PAS such as smoking [22] which may act as unmeasured confounders, though their roles in the pathogenesis of PAS are still under investigation and healthy participants without CAD. Therefore, further longitudinal studies are required to establish the relationship between serum ANGLPT3 level with PAS in patients with CAD.

## 5. Conclusions

The present study demonstrated that fasting serum ANGLPT3 level and older age was positively correlated with increased PAS in patients with CAD. Further prospective studies are required to clarify the causal relationship.

## Figures and Tables

**Table 1:** Clinical variables of the 95 coronary artery disease patients with or without peripheral arterial stiffness.

Characteristics	All Participants (*n* = 95)	Control Group (*n* = 78)	PAS Group (*n* = 17)	*p*-Value
Age (years)	65.20 ± 8.87	64.28 ± 8.86	69.41 ± 7.80	0.030 *
Height (cm)	161.61 ± 8.08	162.09 ± 7.78	159.41 ± 9.27	0.217
Body weight (kg)	69.05 ± 12.08	69.19 ± 12.49	68.41 ± 10.33	0.812
Body mass index (kg/m^2^)	26.35 ± 3.55	26.22 ± 3.60	26.93 ± 3.34	0.459
Systolic blood pressure (mmHg)	131.46 ± 16.74	129.54 ± 15.32	140.29 ± 20.42	0.016 *
Diastolic blood pressure (mmHg)	72.94 ± 10.29	72.82 ± 10.15	73.47 ± 11.21	0.815
Left baPWV (m/s)	15.06 ± 3.30	14.02 ± 2.42	19.86 ± 2.43	<0.001 *
Right baPWV (m/s)	15.13 ± 3.53	13.98 ± 2.50	20.45 ± 2.54	<0.001 *
Total cholesterol (mg/dL)	168.65 ± 35.69	167.95 ± 36.38	171.88 ± 33.14	0.683
Triglyceride (mg/dL)	113.00 (89.00–164.00)	116.00 (92.75–172.00)	97.00 (69.00–153.00)	0.310
HDL-C (mg/dL)	45.19 ± 12.54	44.60 ± 11.57	47.88 ± 16.44	0.331
LDL-C (mg/dL)	97.78 ± 27.06	97.10 ± 26.62	100.88 ± 29.67	0.604
Fasting glucose (mg/dL)	111.00 (97.00–137.00)	105.50 (96.75–131.25)	137.00 (113.50–200.00)	0.008 *
Blood urea nitrogen (mg/dL)	16.00 (13.00–19.00)	16.00 (13.00–18.25)	17.00 (13.50–23.50)	0.225
Creatinine (mg/dL)	1.00 (0.90–1.30)	1.00 (0.90–1.20)	1.30 (0.90–1.50)	0.122
eGFR (mL/min)	68.84 ± 18.64	72.21 ± 17.24	58.93 ± 21.41	0.070
Total calcium (mg/dL)	9.11 ± 0.36	9.10 ± 0.34	9.15 ± 0.48	0.665
Phosphorus (mg/dL)	3.51 ± 0.54	3.52 ± 0.56	3.45 ± 0.46	0.624
iPTH (pg/mL)	46.70 (34.30–66.10)	46.30 (34.30–70.20)	47.50 (33.35–56.15)	0.738
ANGPTL3 (ng/mL)	260.38 (155.31–336.19)	208.41 (146.51–301.83)	347.86 (247.89–493.74)	0.001 *
C-reactive protein (mg/dL)	0.19 (0.14–0.26)	0.18 (0.14–0.23)	0.27 (0.18–0.50)	0.002 *
Female, *n* (%)	24 (25.3)	19 (24.4)	5 (29.4)	0.664
Diabetes, *n* (%)	41 (43.2)	28 (35.9)	13 (76.5)	0.002 *
Hypertension, *n* (%)	77 (81.1)	61 (78.2)	16 (94.1)	0.129
ACE inhibitor use, *n* (%)	28 (29.5)	23 (29.5)	5 (29.4)	0.995
ARB use, *n* (%)	37 (38.9)	28 (35.9)	9 (52.9)	0.192
β-blocker use, *n* (%)	55 (57.9)	45 (57.7)	10 (58.8)	0.932
CCB use, *n* (%)	32 (33.7)	24 (30.8)	8 (47.1)	0.198
Statin use, *n* (%)	69 (72.6)	59 (75.6)	10 (58.8)	0.159
Fibrate use, *n* (%)	21 (22.1)	16 (20.5)	5 (29.4)	0.423
Aspirin, *n* (%)	72 (75.8)	61 (78.2)	11 (64.7)	0.239
Clopidogrel, *n* (%)	25 (26.3)	20 (25.6)	5 (29.4)	0.749

Values for continuous variables are shown as mean ± standard deviation after analysis by Student’s *t*-test; variables not normally distributed are shown as median and interquartile range after analysis by the Mann–Whitney U test; categorical variables are presented as number (%) and analysis after analysis by the chi-square test. * *p* < 0.05 was considered statistically significant. PAS, peripheral arterial stiffness; baPWV, brachial-ankle pulse wave velocity; HDL-C, high-density lipoprotein cholesterol; LDL-C, low-density lipoprotein cholesterol; eGFR, estimated glomerular filtration rate; iPTH, intact parathyroid hormone; ANGPTL3, angiopoietin-like protein 3; ACE, angiotensin-converting enzyme; ARB, angiotensin-receptor blocker; CCB, calcium-channel blocker.

**Table 2:** Multivariable logistic regression analysis of the factors correlated to peripheral arterial disease among the 95 coronary artery disease patients.

Variables	Odds Ratio	95% Confidence Interval	*p*-Value
Angiopoietin-like protein 3, 1 ng/mL	1.004	1.000–1.007	0.041 *
Age, 1 year	1.138	1.028–1.259	0.012 *
Diabetes, present	3.699	0.737–18.574	0.112
C-reactive protein, 0.1 mg/dL	1.196	0.956–1.497	0.118
Fasting glucose, 1 mg/dL	1.011	0.997–1.025	0.119
Systolic blood pressure, 1 mmHg	1.028	0.986–1.072	0.189

ANGPTL3, angiopoietin-like protein 3. * *p* < 0.05 was considered statistically significant in the multivariate logistic regression analysis (adopted factors: diabetes, age, systolic blood pressure, fasting glucose, C-reactive protein, and ANGPTL3).

## Supplementary Materials

The following are available online at https://www.mdpi.com/article/10.3390/medicina57101011/s1. Table S1: Correlation of left brachial-ankle pulse wave velocity and clinical variables by simple or multivariable linear analyses among 95 patients with coronary artery disease. Table S2: Correlation of right brachial-ankle pulse wave velocity and clinical variables by simple or multivariable linear analyses among 95 patients with coronary artery disease.
