# Supplementary material for: Serum Angiopoietin-like Protein 3 Level Is Associated with Peripheral Arterial Stiffness in Patients with Coronary Artery Disease
Source: Medicina (Kaunas). 2021 Sep 25;57(10):1011. doi: 10.3390/medicina57101011 (PMC8538502; doi:10.3390/medicina57101011)
Supplement: Supplementary file 1 [file medicina-57-01011-s001.zip › medicina-1352943-supplementary.pdf]

Table S1. Correlation of left brachial-ankle pulse wave velocity and clinical variables by simple or multivariable linear analyses among 95 patients with coronary artery disease.

**Variables**	**Left brachial-ankle pulse wave (m/s)**				
	**Simple Linear Regression**		**Multivariable Linear Regression**		
	r	***p* Value**	**Beta**	**Adjusted R^2 change**	***p* Value**
Female	0.152	0.142	—	—	—
Diabetes mellitus	0.284	0.005*	0.236	0.040	0.006*
Hypertension	0.084	0.420	—	—	—
Age (years)	0.278	0.006*	0.190	0.025	0.036*
Body mass index (kg/m^2)	-0.053	0.609	—	—	—
Systolic blood pressure (mmHg)	0.209	0.042*	—	—	—
Diastolic blood pressure (mmHg)	0.048	0.643	—	—	—
Total cholesterol (mg/dL)	0.045	0.666	—	—	—
Log-triglyceride (mg/dL)	0.015	0.886	—	—	—
HDL-C (mg/dL)	0.143	0.168	—	—	—
LDL-C (mg/dL)	0.007	0.944	—	—	—
Log-glucose (mg/dL)	0.191	0.064	—	—	—
Log-BUN (mg/dL)	0.091	0.380	—	—	—
Log-creatinine (mg/dL)	0.180	0.081	—	—	—
eGFR (mL/min)	-0.291	0.004*	—	—	—
Total calcium (mg/dL)	0.090	0.384	—	—	—
Phosphorus (mg/dL)	0.048	0.643	—	—	—
Log-iPTH (pg/mL)	-0.004	0.968			
Log-CRP (mg/dL)	0.388	< 0.001*	0.265	0.067	0.003*
Log-ANGPTL3 (ng/mL)	0.417	< 0.001*	0.286	0.165	0.002*

Data of triglyceride, glucose, BUN, creatinine, iPTH, C-reactive protein and ANGPTL3 levels showed skewed distribution, and therefore were log-transformed before analysis. Analysis of data was done using the simple linear regression analyses or multivariable stepwise linear regression analysis (adapted factors were diabetes mellitus, age, systolic blood pressure, eGFR, log-CRP and log-ANGPTL3). HDL-C, high-density lipoprotein cholesterol; LDL-C, low-density lipoprotein cholesterol; BUN, blood urea nitrogen; eGFR, estimated glomerular filtration rate; iPTH, intact parathyroid hormone; ANGPTL3, angiopoietin-like protein 3. *$p < 0.05$ was considered statistically significant.

Table S2. Correlation of right brachial-ankle pulse wave velocity and clinical variables by simple or multivariable linear analyses among 95 patients with coronary artery disease..

**Variables**	**Right brachial-ankle pulse wave (m/s)**				
	**Simple Linear Regression**		**Multivariable Linear Regression**		
	r	***p* Value**	**Beta**	**Adjusted R^2 change**	***p* Value**
Female	0.113	0.278	—	—	—
Diabetes mellitus	0.259	0.011*	0.229	0.046	0.008*
Hypertension	0.072	0.488	—	—	—
Age (years)	0.292	0.004*	0.254	0.048	0.004*
Body mass index (kg/m^2)	-0.010	0.921	—	—	—
Systolic blood pressure (mmHg)	0.221	0.031*	—	—	—
Diastolic blood pressure (mmHg)	0.042	0.689	—	—	—
Total cholesterol (mg/dL)	0.104	0.318	—	—	—
Log-triglyceride (mg/dL)	0.001	0.998	—	—	—
HDL-C (mg/dL)	0.190	0.065	—	—	—
LDL-C (mg/dL)	0.062	0.552	—	—	—
Log-glucose (mg/dL)	0.205	0.047*	—	—	—
Log-BUN (mg/dL)	0.022	0.833	—	—	—
Log-creatinine (mg/dL)	0.126	0.223	—	—	—
eGFR (mL/min)	-0.237	0.021*	—	—	—
Total calcium (mg/dL)	0.067	0.517	—	—	—
Phosphorus (mg/dL)	-0.008	0.936	—	—	—
Log-iPTH (pg/mL)	-0.023	0.823			
Log-CRP (mg/dL)	0.403	< 0.001*	0.270	0.083	0.003*
Log-ANGPTL3 (ng/mL)	0.411	< 0.001*	0.299	0.160	0.001*

Data of triglyceride, glucose, BUN, creatinine, iPTH, C-reactive protein and ANGPTL3 levels showed skewed distribution, and therefore were log-transformed before analysis. Analysis of data was done using the simple linear regression analyses or multivariable stepwise linear regression analysis (adapted factors were diabetes mellitus, age, systolic blood pressure, log-glucose, eGFR, log-CRP and log-ANGPTL3). HDL-C, high-density lipoprotein cholesterol; LDL-C, low-density lipoprotein cholesterol; BUN, blood urea nitrogen; eGFR, estimated glomerular filtration rate; iPTH, intact parathyroid hormone; ANGPTL3, angiopoietin-like protein 3. *$p < 0.05$ was considered statistically significant.
